# Deep learning assisted cancer disease prediction from gene expression data using WT-GAN

**DOI:** 10.1186/s12911-024-02712-y

**Published:** 2024-10-24

**Authors:** U. Ravindran, C. Gunavathi

**Affiliations:** 1 School of Computer Science Engineering and Information Systems, Vellore Institute of Technology, Vellore, India; 2 School of Computer Science and Engineering, Vellore Institute of Technology, Vellore, India

**Keywords:** Deep learning methods, Microarray gene expression data, WT-GAN, Machine learning methods

## Abstract

Several diverse fields including the healthcare system and drug development sectors have benefited immensely through the adoption of deep learning (DL), which is a subset of artificial intelligence (AI) and machine learning (ML). Cancer makes up a significant percentage of the illnesses that cause early human mortality across the globe, and this situation is likely to rise in the coming years, especially when non-communicable illnesses are not considered. As a result, cancer patients would greatly benefit from precise and timely diagnosis and prediction. Deep learning (DL) has become a common technique in healthcare due to the abundance of computational power. Gene expression datasets are frequently used in major DL-based applications for illness detection, notably in cancer therapy. The quantity of medical data, on the other hand, is often insufficient to fulfill deep learning requirements. Microarray gene expression datasets are used for training procedures despite their extreme dimensionality, limited volume of data samples, and sparsely available information. Data augmentation is commonly used to expand the training sample size for gene data. The Wasserstein Tabular Generative Adversarial Network (WT-GAN) model is used for the data augmentation process for generating synthetic data in this proposed work. The correlation-based feature selection technique selects the most relevant characteristics based on threshold values. Deep FNN and ML algorithms train and classify the gene expression samples. The augmented data give better classification results (> 97%) when using WT-GAN for cancer diagnosis.

## Introduction

**Fig. 1:**
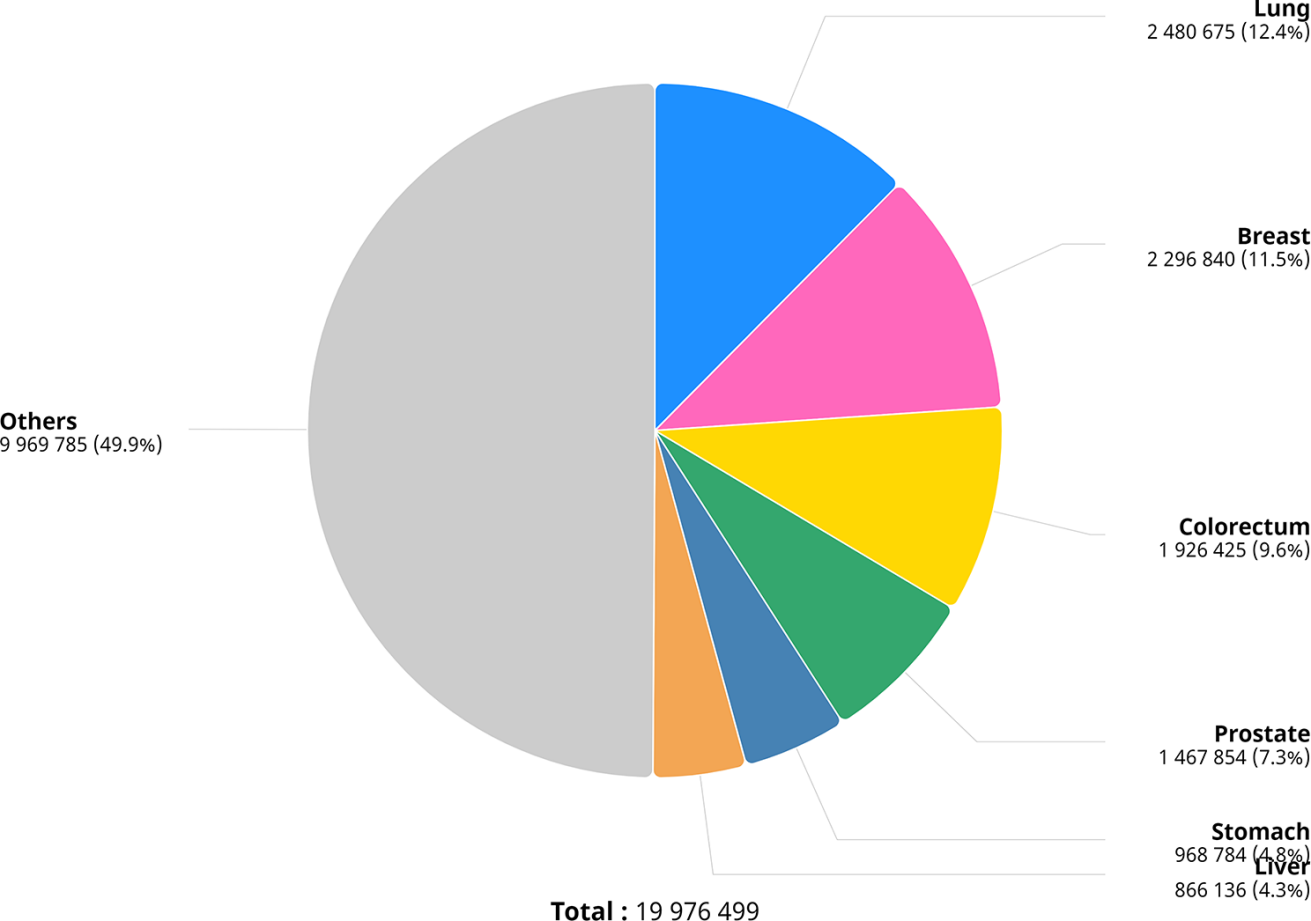
Different types of cancer across the globe

Cancer is a malignant condition that affects individuals of all ages, from all walks of life and geographical areas. Figure 1 shows the different types of cancer in the world from the Global Cancer Observatory (GCO) [1]. Cancer is one of the main causes of death globally, with an estimated 10 million fatalities from the disease. Cancer is responsible for one out of every six deaths worldwide. According to the statistics of new cancer cases, female breast cancer has overtaken lung cancer as the most common type of cancer diagnosed, with around 11.7% of new cases, followed by lung cancer at 11.4%, colorectal cancer at 10.4%, prostate cancer at 7.3%, and stomach cancer at 5.6%. According to the cancer death statistics, lung cancer continues to be the major cause at around 18%, followed by colorectal cancer at 9.4%, liver cancer at 8.3%, stomach cancer at 7.7%, and female breast cancer at 6.9% [2].

Microarray Gene Expression Cancer Data (MGECD) is better suited for cancer categorization and ultimately aiding qualitative research. The MGECD is derived from a chromosome in a human cell. The collection of information on the various levels of gene expression occurs in three stages. The isolation phase is the initial stage in the process of isolating messenger ribonucleic acid (mRNA). Reverse transcription is the second stage of the process to obtain the complementary deoxyribonucleic acid (cDNA) fragments from the mRNA using reverse transcription-polymerase chain reaction (RT-PCR). The hybridization phase is the third stage in which cDNA is labeled with fluorescent tags and hybridized with probes to express gene information on arrays. Microarray technology enables the simultaneous monitoring of numerous gene expression levels. Typically, gene expression data is used to increase prediction accuracy in cancer categorization [3].

Individuals are affected by many forms of cancer in a variety of circumstances. As a result, the situation must be closely monitored. The quantity of cancer records (data) that researchers can access has grown tremendously as a result of technological advancements. However, drawing accurate conclusions from such information is a difficult undertaking. Making use of artificial intelligence (AI) techniques makes this a reality. It is now possible to examine the patterns in huge and complicated datasets using AI techniques. There is no limit to the quantity of data that deep learning models can process. Machine learning (ML) algorithms can find significant links between genetic or molecular markers and various cancers by detecting hidden patterns and relationships in the data, improving classification accuracy. Traditional machine learning methods, such as support vector machines (SVM), random forests (RF), logistic regression (LR), and decision trees (DT), are used to train and classify the data samples. Deep learning (DL) methods are heavily used in the field of diagnostic genomics [4]. DNA microarray data is a kind of high-throughput genetic analysis. It is to analyze and evaluate the different gene expression levels simultaneously to provide information about the condition of cells. The significant variations in the microarray gene expression levels lead to the identification of appropriate cancer cells and the selection of the best approach for cancer therapy. A deep-learning approach inspired by neural networks is incorporated as part of the research for the prediction of different cancer categories through MGECD. Traditional deep learning methods, such as convolutional neural networks (CNN), recurrent neural networks (RNN), feed-forward neural networks (FFN), and auto-encoders (AE), were analyzed in MGECD for the classification of cancer diseases and their types [5].

In the field of cancer research, the MGECD’s dimensionality burden is the most challenging problem to solve. It includes the most gene features in the dataset, despite having the fewest samples. Since the priority of the study is centered on the prediction accuracy of cancer disease, the gene dataset’s influence is a significant concern because a dataset’s effectiveness is primarily dependent on the data that is fed into it. To cope with the large datasets, to investigated several methods and methodologies. Considering this aspect as a reference, sample size can be determined using several distinct approaches [6]. As described by MGECD, representative sample size concerns the proportion of arrays needed to achieve the optimal subdivision of the predefined metric at the specified conventional impactful dimensions. Several tests have been performed briefly using sparse and informal datasets. Considering that the formulated sample size has not altered over time, the impacts of identifying facts are more likely to be reduced when processing the sparse data. However, since the current approach relies on a minuscule sample, the findings are not as accurate as they might be [7].

The MGECDs are mainly used to identify the specific cancer disease. The gene data consists of a huge number of features (genes) and a small number of samples. Synthetic data is similar to the actual data that is generated to anticipate the cancer disease with a better outcome. Investigate several approaches and methods for data augmentation procedures to expand the synthetic dataset. Two of the most prominent methods for producing AI-generated material are variational autoencoders (VAE) and generative adversarial networks (GAN). VAEs are autoencoders that specialize in dimensionality reduction and minimize the error between reconstructed data and original data. VAEs are highly effective at reconstructing input data from the encoded latent representation. It is an unsupervised learning algorithm mainly used to detect anomalies [8]. Another generative model is generative adversarial networks (GAN), which specialize in producing sharp, realistic data due to their innovative adversarial mechanisms.

Generative adversarial networks (GAN) are a data augmentation technique that was developed to handle the generative modeling challenge. The min-max two-player game inspired the basic concept of GAN. The two main components of a traditional GAN are the generator G, which mimics the true data distribution, and the discriminator D, which tries to tell the difference between real and fake data. The goal of the generative model is to analyze a set of training samples and determine the probability distribution that developed them. The GAN network is used to generate realistic samples that are similar to the original samples in the datasets. The traditional GAN network aims to generate synthetic image samples and minimize the loss value of the generator network. The discriminator is not providing enough information to generate the fake samples. The tabular GAN is used to create numerical samples instead of image samples. To calculate the similarity between two probability distributions in the tabular GAN model, Jensen-Shannon divergence (JSD) is utilized. The gradient update is unstable, and the difference in penalties for diversity and accuracy leads to mode collapse, i.e., a lack of diversity [9].

To obtain the necessary data samples to overcome mode collapse, to present a WT-GAN technique to handle this problem. WT-GAN is used to generate numerical data instead of image data, and the loss value is calculated using the Wasserstein distance. The Wasserstein loss is intended to prevent vanishing gradients even when the discriminator is optimally trained. In Discriminator, the training simply attempts to increase the output for real data over fake data. The correlation coefficient (CC) is used to identify significant aspects of gene data. It measures the degree of relationship among variables of interest based on a threshold value. For the training and classification of augmented and non-augmented gene expression data, deep learning and machine learning algorithms are utilized.

The WT-GAN technique will be used in this research work to improve the MGECD’s performance. To generate numerical synthetic samples, the proposed method is WT-GAN along with a correlation coefficient filter for selecting the most relevant features. The proposed technique produces considerable improvements in outcomes. This research work comprises five sections: Sect. 1 includes the essential introduction of the proposed model. Section 2 elaborates on the most relevant studies that focus on the prediction of cancer disease. Section 3 describes the methodology of the proposed model. Section 4 discusses the results of the proposed model with other techniques, and Sect. 5 presents the future work and conclusion of the proposed model.

## Related work

New advances in DL have opened up a new frontier in the feature extraction, classification, and prediction of target classes. In terms of prognosis, DL networks are distinct from conventional ML techniques. Thus, this section covers the major relevant works of literature that deal with the prediction of cancer diseases.

Chaudhari et al. [10] utilized a modified generative adversarial model to generate the data samples with multivariate noise and a Gaussian distribution. The MG–GAN network was used to reach the saddle point quicker since the created data is in feature space. This model improved classification accuracy by 18.8% when compared to KNN and 11.9% when compared to traditional GAN. This model was suitable for sensitive data applications; the error value was dramatically reduced from 0.6978 to 0.0082.

On a publicly accessible dataset called “Kent Ridge Biomedical Repositories”, Huynh et al. [11] generated the new data samples using the GAN approach to classify healthy and tumor samples. The data were characterized by around 20 datasets, and they were used for multi-class and binary-class categorization. The classification performance of GAN-SVM compared to KNN, RF, DT, and Linear SVM. The GAN-SVM produced better classification results for the 15 gene expression datasets.

Using an ensemble feature selection technique, Wang et al. [12] increased the discrimination and reliability of the finally gathered features. They applied sampling approaches to obtain numerous sampled datasets for selecting the subset of features from the base feature selector. They used aggregation strategies to combine them into a single set. This approach achieved better stability and also ensured better classification performance.

Basavegowda et al. [13] developed a 7-layer deep feed-forward neural network to categorize the microarray gene expression data into a set of classifications for further cancer diagnosis. The gene expression dataset is downloaded from the “ELVIRA Biomedical Dataset Repository”. To reduce the dimensionality of the data, they used the principal component analysis technique. For selecting the relevant feature values, they utilized the Min-Max approach. To evaluate the loss value, a cross-entropy is employed and an adaptive moment estimate is used for optimization. The proposed model achieved better recall and accuracy values compared to existing traditional methods.

Khalifa et al. [14] proposed an innovative deep-learning strategy that used binary particle swarm optimization along with a decision tree (BPSO-DT) for retrieving the optimal features from the RNA sequence dataset, which they converted into two-dimensional data. The augmentation technique was applied to make the 2D data five times larger for improved accuracy results and to prevent the overfitting problem. CNN architecture was used to classify the five different types of tumors. The proposed model achieved a testing accuracy of 96.9%.

Abdelhalim et al. [15] generated skin lesion images using self-attention progressive-growing GAN (SP-GAN). A CNN model was used to detect melanoma, which is difficult with traditional GANs due to the high quality of the images and the variety of skin lesions in shape, size, and location. In SPGAN, it aggregates all the information in the feature locations. At the time of the discriminator process, it detailed the related features in the image. To improve the stat ability while generating the lesion images, the Two-Timescale Update Rule (TTUR) was applied. This model improved the sensitivity value to 5.6% for non-augmented data and 2.5% for augmented data. In the melanoma class, the sensitivity improved by 13.8% for non-augmented data and 8.6% for augmented data.

Chaudhari et al. [16] proposed an enhanced form of the K-nearest neighbor (KNN) rule. To obtain synthetic samples, they employed counting filters for effectively skewed data, the Euclidean distance for estimating the distance between its neighbors, and the best mean value from the KNN for each target sample. SVM, J48, and DNN are a few of the techniques used to further classify the data produced by these methods. The generated samples by the enhanced technique have higher recall values than the traditional implementation, and it also ensures data sensitivity. Improved KNN produced better classification accuracy as compared to the traditional KNN approach; it improved by 7.72% for augmented data and 16% while processing the raw data before augmentation.

Xiao et al. [17] suggested the WGAN (Wasserstein GAN) model, which gives a dependable training progress indicator and thoroughly investigates data features. The WGAN creates new samples among the minority class and overcomes the data imbalance problem. The WGAN method solves the unbalanced learning problem of the RNA sequence dataset. For breast cancer, they achieved an accuracy value of 98.3%, precision of 100%, recall of 96.67%, and an F1-Score of 89.31%. For stomach cancer, they achieved an accuracy value of 96.67%, precision of 100%, recall of 93.33%, and an F1-Score of 96.55%. For lung cancer, they achieved an accuracy value of 96.67%, precision of 100%, recall of 93.33%, and an F1-Score of 95.55%.

Park et al. [18] proposed a method for learning genetic networks from multi-omics data. Instead of identifying specific biomarkers for cancer prognosis, they proposed learning the network of prognostic genes. This method employs a GAN network in which the generator learns the distributions of gene expression (mRNA), DNA methylation, copy number variation (CNV), and single nucleotide polymorphism (SNP) data from cancer samples. This proposed model was utilized on seven types of cancer datasets, and it predicted higher accuracy. The AUC curve was improved by 4% when compared to existing methods specifically; the AUC curve was improved by 27.9% in the pancreatic adenocarcinoma data.

Wei et al. [19] utilized a generative adversarial model for non-target cancer data to aid in target generator training. The reconstruction loss is used to improve model stability and sample quality. Additionally, they improve classification accuracy. In terms of classification accuracy, the proposed model achieved 92.6%, which is 7.6% greater than its performance without any generated data. Almarzouki et al. [20] used a wrapper-based feature selection technique, the Artificial Bee Technique (ABC), to retrieve the most relevant information from the dataset. This method produces a subset of each feature created by the observers. The goal is to reduce the execution time while selecting the fewest genes and improve the classification accuracy. The proposed CNN model was used to predict cancer and non-cancer samples; it achieved 96.43% accuracy. Al-Obeidat et al. [21] utilized a two-stage unsupervised feature selection for classification of cancer samples. In the first stage, the author aggregates the three feature selection techniques, namely correlation-based, spectral-based, and principal component analysis. It is an auto-encoder-based clustering method that was used to extract the feature sets. Traditional algorithms like SVM, KNN, and RF were used for classification purposes.

To enhance the classification accuracy of the breast cancer gene data, Adebiyi et al. [22] utilized machine learning classifiers like RF and SVM along with the feature extraction technique linear discriminant analysis (LDA). They achieved 96.4% and 95.6% accuracy while using SVM and RF classifiers along with the LDA technique. Arowolo et al. [23] compared the feature extraction techniques such as supervised Partial Least Square (PLS) and unsupervised Principal Component Analysis (PCA) for retrieving the most significant features before classifying the colon cancer data. The SVM was used to classify the gene expression data. The SVM with the PLS algorithm achieved a better accuracy of 95.2% when compared to the SVM with PCA, which achieved only 82.3%. For analyzing and predicting colon cancer disease, Arowolo et al. [24] utilized machine learning algorithms like SVM, RF, and KNN with PCA feature extraction techniques. The RF classifier achieved better results when compared to SVM and KNN. It achieved around 96% accuracy with PCA and 84% accuracy without PCA. Mahesh et al. [25] proposed a hybrid approach, Particle Swarm Optimization (PSO) with Ant Lion Optimization (ALO), to predict leukemia cancer using microarray gene data. The SVM classifier was utilized to classify the gene expression data after selecting the relevant features. The proposed method achieved a better classification accuracy of 87.88%. Cusworth et al. [26] solved the class imbalance problem in the gene expression dataset using the GAN method. The GAN model significantly improved an AUC value greater than 0.65, but the traditional augmentation techniques like the synthetic minority oversampling technique (SMOTE) and random oversampling (RO) achieved less than 0.65.

The major related work in the literature carries the traditional GAN architecture for image data augmentation. The traditional Tabular-GAN is used to generate numerical data rather than image data. In the tabular GAN architecture, Jensen Shannon (JS) divergence is utilized, but the gradient update is unstable, which leads to mode collapse. To overcome the problem, the Wasserstein distance is calculated so that training variables become more stable. It reduces the oscillations and instability frequently seen with JS divergence. In the proposed model, the Wasserstein Tabular Generative Adversarial Network (WT-GAN) is used to generate the synthetic samples for gene expression data. Table 1 describes the related work based on cancer disease prediction using neural networks.

**Table 1:** Neural network-based Cancer Prediction models

Ref	Network Type	Predictor	Cancer Type	Samples	Classes	Metrics
[10]	MGGAN	KNN DNN	Lung Prostate Leukemia Breast Colon	192 171 66 162 202	Two	Accuracy Precision
[11]	Traditional GAN	SVM RF DT LSVM	20 different datasets	50–100 samples from each dataset	Two Three Four Five Nine	Accuracy
[12]	Ensemble Feature Selection	NB KNN SVM RF	SRBCT Colon DLBCL Leukemia	83 62 77 72	Four Two Two Three	Mean Standard Deviation
[13]	Traditional GAN	DFNN	CNS Colon Leukemia Prostate Ovarian Breast Lung	60 62 72 102 253 97 96	Two	Accuracy Recall
[14]	BPSO-DT	CNN	BRCA KIRC LUAD LUSC UCEC	878 537 162 240 269	Two	Accuracy Precision Recall F1-Score
[15]	SPGAN	CNN	ALL	10015images	Four	Sensitivity
[16]	Improved KNN	SVM DNN	Lung Prostate Leukemia Breast Colon	256 228 88 216 262	Two	Accuracy Sensitivity
[17]	WGAN	SVM	LUAD STAD BRCA	162 271 878	Two	Accuracy Precision Recall F1-Score
[18]	Traditional GAN	FNN	BRCA LAML LIHC LUAD PAAD STAD LGG	154 96 134 105 44 45 125	Two	p-value
[19]	Gene GAN	CNN	Lung Breast Prostate Colon	137 263 123 98	Two	Accuracy
[29]	Artificial Bee Colony	CNN	Renal Lung Brain	580 1185 584	Two	Accuracy Precision Recall
[21]	PCA Correlation Spectral	SVM KNN RF	Leukemia DLBCL Colon Lung Prostate	72 77 62 181 136	Two	Accuracy Precision Recall F1-Score
[22]	LDA	SVM RF	Breast	569	Two	Accuracy Sensitivity Specificity Precision F1-Score
[23]	PLS PCA	SVM	Colon	62	Two	Accuracy Sensitivity Specificity Precision AUC
[24]	PCA	SVM RF KNN	Colon	5000	Two	Accuracy Sensitivity Specificity
[25]	PSO-ALO	SVM	Leukemia	72	Two	Accuracy
[26]	Random Sampling, SMOTE, GAN	GAN	4 different datasets	400–500 samples from each dataset	Two	AUC

## Method

### Proposed architecture

Minimal training sets lead to a trained model that is too closely tied to its original data (overfitting) and has low generalizability. Expansion of the training data has been extensively adopted to prevent such overfitting (low generalization) issues, usually referred to as data augmentation. It further enables a more extensive connectivity system to be utilized without severe co-linearity. Data augmentation is frequently employed when a minor modification is made to the samples of source datasets and additional samples are generated synthetically. Data Augmentation using WT-GAN is part of the research strategy.

**Fig. 2:**
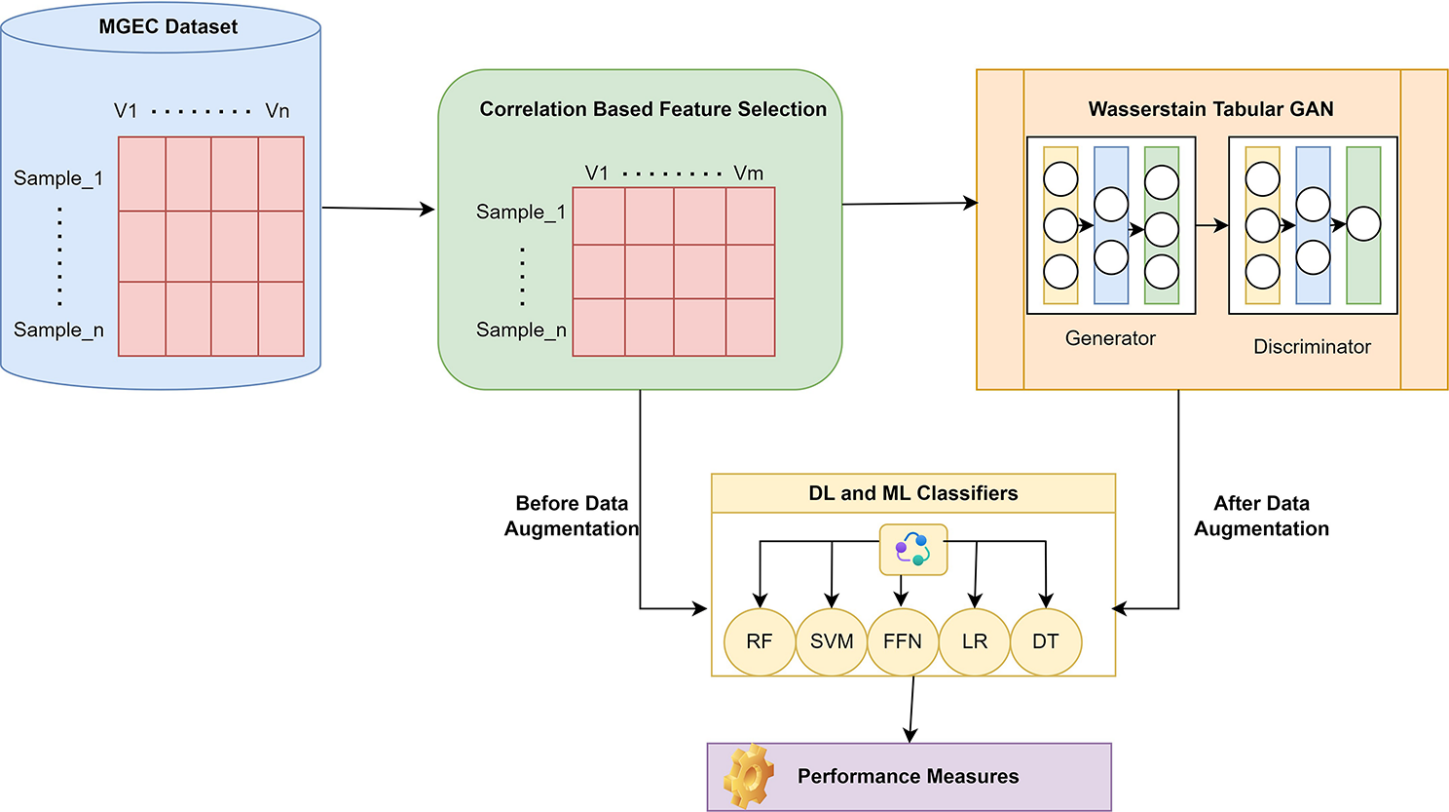
Architecture of the Proposed Model

The Microarray gene expression cancer data is taken as input for this research work, and performance is evaluated based on the augmentation strategy. The filter-based feature selection technique is applied to select the important features in the gene expression data. The selected features are classified using traditional classifiers. Data Augmentation Strategy WT-GAN is used to generate fake data; it is similar to the original data to solve the dimensionality problem in the gene expression dataset. So, this type of data improvement strategy aims to provide the final classifier training set with highly refined, suitable data samples to evaluate the performance of the proposed model based on the augmented and non-augmented data and to ensure the quality of the fake data generated. Figure 2 represents the architecture of the proposed model.

### Correlation-based feature selection

In this research, feature selection is carried out via a correlation approach. In a correlation analysis, the correlation coefficient is the precise metric that expresses the probability that two variables have a linear relationship with each other. Such an approach uses a correlation-based computation mechanism to calculate the correlation coefficients (*CC*) of different gene features. In feature selection, the correlation technique is applied to select the most relevant features based on the threshold value. The correlation value of a variable tells how the specific variable is related to the target variable [27]. When a variable’s relationship with its target is “1,” it indicates that the two variables are highly connected and their values are directly proportional, meaning that when the variable’s value rises, so does the target’s. When a variable’s relationship with its target is “-1,” it indicates that the two are not highly connected and their values are indirectly proportional, meaning that when the variable’s value rises, the target’s value falls. Selecting the highly correlated features for classification and dropping the less correlated features does not have much impact on the classification results [28].

It overcomes the restrictions posed by univariate filter techniques, which do not consider the interactions of gene components (features). For example, correlation factors can be applied to examine the connection between different pairs of gene expressions. Pearson’s CC, also known as correlated indices in analytics, can be used to define a linear connection among varying gene pairs. The term “correlation” refers to an estimation of the degree of relevance or association between two attributes. In linear relationships, the CC between two traits is ± 1. If the characteristics are uncorrelated, the CC is zero. The following expression (1) from [29] can be applied to determine the linear CC (R) for a specific set of gene variables.

1 $$\eqalign{{\bf{\it{R}}}\, = \, & \sum {\left[ {\left( {{{\bf{\it{V}}}_i}\, - \,{{{\bf{\it{\bar V}}}}_i}} \right){\mkern 1mu} \left( {{{\bf{\it{U}}}_i}\, - \,{\bf{\it{\bar U}}}} \right)} \right]} \cr & /\sqrt {\sum {\left[ {{{\left( {{{\bf{\it{V}}}_i}\, - \,{{{\bf{\it{\bar V}}}}_i}} \right)}^2} \cdot {{\left( {{{\bf{\it{U}}}_i}\, - \,{{{\bf{\it{\bar U}}}}_i}} \right)}^2}} \right]} } \cr}$$

Pearson’s correlation coefficient (PCC) was utilized to identify the important features of the gene data. This is indeed a computational estimate of the linear association between two variables, U and V. The following are the restrictions imposed by R on the variables, which are conditionally stated as [-1 ≤ *R* ≤ 1]. That is, when R more closely approaches 1, it signifies the positive correlation; conversely, closer to zero or exactly 0 signifies they are non-linear in relationships; and ultimately, any negative values signify the negative correlation. Pearson’s correlation was utilized to determine the degree of association among the various concerning features in the suggested model. Commonly, features would be selected if the score was greater than the cutoff point (ζ = 0.5). The selection gets completed whenever the feature count equals (n-log n). Each attribute is listed in a substantially reduced order of importance. Finally, the feature-to-feature interaction procedure is performed to eliminate the superfluous features.

The principal objective of this research is to eliminate non-essential features. Initially, some base paradigms regarding the selection procedures are referred to in [30]. Here, the overall feature set is denoted as *|F|*, and *f*_*i*_ indicates the gene feature. Thus, the relevance *r*_*i*_ can be attained via *r*_*i*_ = *[|F| - {f*_*i*_*}]*, and the conditional likelihood (*L*) of cancer class (*k*) to a given *f*_*i*_ is expressed as,

2 $$\:{\textbf{f}}_{\textbf{i}}=\left\{\begin{array}{c}relevant,\:\:\:\:\:\:\:\exists\:{\textbf{r}}_{\textbf{i}}^{\prime}\subseteq\:{\textbf{r}}_{\textbf{i}},\:\left(\textbf{i}.\textbf{e}\right)L\left[\langle\textbf{k}|{\textbf{f}}_{\textbf{i}},{\textbf{r}}_{\textbf{i}}^{\prime}\rangle\right]\ne\:L\left[\langle\textbf{k}|{\textbf{r}}_{\textbf{i}}^{{\prime}}\rangle\right]\\\:irrelevant,\:\:\:\:\:\:\:\:\:\:\:\:\:\:\:\:\:\:\:\:\:\:\:\:\:\:\:\:\:\:\:\:\:\:\:\:\:\:\:\:\:\:\:\:\:\:\:\:\:\:\:\:\:Otherwise\end{array}\right.$$

According to Eq. (2), it is only meaningful if eliminating a *f*_*i*_ from a *|F |*reduces the predictive ability. It can be seen from the statement that a *f*_*i*_ can be found to be relevant based on two factors,

- It is significantly associated with the intended target class.
- Together with various *f*_*i*_, it creates a subset, and this *f*_*i*_-subset is highly associated with the target notion.

Thus, if *f*_*i*_ is significant due to the second rationale, feature interaction occurs.

### Wasserstein Tabular GAN

The fake data samples (synthetic) were created using the GAN. Thus, it is vital to understand the GAN’s fundamental functioning mechanism. Dual adversary networking approaches *(g(f)*, *d(f))* are used in GANs, where one network (generator *g(f)*) undergoes essential training to construct synthetic data based on the discriminator’s *d(f)* feedback while the other trains to differentiate the synthetic data from the actual (primitive) ones. The generator’s purpose is to optimize a cost (significance) value *Ψ (g*,* d)*, but the discriminator’s objective is to elevate it.

Even though this method may provide excellent results, the process of objectively assessing generative models is currently unclear. Approaches that produce excellent sample data may have a relatively low probability (the likelihood that such a system is comparable to the learning information). In contrast, approaches that have a strong probability can produce unsatisfactory data samples.

Thus, the primary augmentation technique is the WT-GAN, which is an improvement of the GAN and offers better consistency. WT-GAN utilizes numerical data to compute the distribution of the original and generated data mathematically instead of image data. Tabular data T contains discrete random variables like D = {n_1_, n_2,,_ ., n_d_} and continuous random variables like C = {n_1_, n_2_,.,n_c_}.These variables have an unknown joint distribution, P(C, D). The goal is to learn a generative model M (C, D) such that samples generated by this model M produce a synthetic table Tsynthesis that can attain the same accuracy on a real test table Ttest as a model learned with data from table T. A random pair of variables i and j in T and Tsynthesis have similar mutual information [31]. Numerical variable preprocessing Using Tanh activation, neural networks may successfully generate the values with a distribution centered on (-1, 1). However, they show that when dealing with multi-modal input, networks fail to generate enough data. They use and train a Gaussian Mixture Model (GMM) to cluster a numerical variable. By preprocessing categorical variables because of their low cardinality, they discovered that the probability distribution may be constructed directly using softmax. However, before being converted to binary variables, categorical variables must be changed to a one-hot-encoding format with noise [32].

The Wasserstein distance is used to compute the distribution of the original and synthetic data instead of the Jensen-Shannon divergences. The Wasserstein distance, also known as Earth Mover’s Distance, is the distance between two probability distributions over a particular region. WD can nevertheless give a logical and consistent estimate of the distance between them. The WD loss is to minimize the loss between the real and fake data to avoid mode collapse and vanish the gradients. As a result, it is ready to address the instability issue that affects the standard GAN.

In Eq. (3), it is shown that computing the Wasserstein distance between two data distributions. The distance between two distributions can be viewed as the “cost” of the best transport plan for moving one distribution’s probability mass until it matches the other. As a result, if we have two potential generative distributions, the Wasserstein distance reveals which one is closer to pdata, even if neither of them overlaps. The JSD employed by the original GAN loss, on the other hand, is unlimited for no overlapping distributions. As a result, WGAN enables powerful discriminators to supply valuable gradient information to the generator even while generating the quality of the samples remains poor, making training more stable. The objective function of the Tabular GAN using Wasserstein distance is shown in Eq. (4) [33].

3 $$\:\varvec{W}\left[{\varvec{\mu\:}}_{\varvec{i}},{\varvec{\mu\:}}_{\varvec{j}}\right]=\underset{\varvec{\rho\:}\in\:\varvec{\phi\:}\left({\varvec{\mu\:}}_{\varvec{i}},{\varvec{\mu\:}}_{\varvec{j}}\right)}{\varvec{Inf}}\left(\left[‖\varvec{a}-\varvec{b}‖\right]\bullet\:{\varvec{E}}_{\left(\varvec{a},\varvec{b}\right)\sim\varvec{\rho\:}}\right)$$

Where, *µ*_*i*_ and *µ*_*j*_ denote the original and generated data distribution, respectively, *φ(µ*_*i*_, *µ*_*j*_*)* represents the possible joint distribution of *ρ(a*,* b)* whose extreme parameters are *µ*_*i*_ and *µ*_*j*_, respectively. *‘φ’* includes all potential distributions of *ρ.*

4 $$\:\varvec{V}\left(\varvec{G},\varvec{D}\right)=\underset{\varvec{Gen}}{\mathbf{min}}\:\underset{\varvec{Dis}}{\mathbf{max}}{\varvec{E}}_{\varvec{x}\leftarrow\:\varvec{p}\varvec{d}\varvec{a}\varvec{t}\varvec{a}}[\varvec{D}\varvec{i}\varvec{s}\left(\varvec{x}\right)-{\varvec{E}}_{\varvec{z}\leftarrow\:\varvec{p}\left(\varvec{z}\right)}[\varvec{D}\varvec{i}\varvec{s}\left(\varvec{G}\varvec{e}\varvec{n}\left(\varvec{z}\right)\right]$$

Where x represents the original distribution and z denotes the generated distribution. The objective function of the generator is to minimize, and that of the discriminator is to maximize. The discriminator provides feedback to the generator, giving them the freedom to generate the data based on the boundary values.

**Fig. 3:**
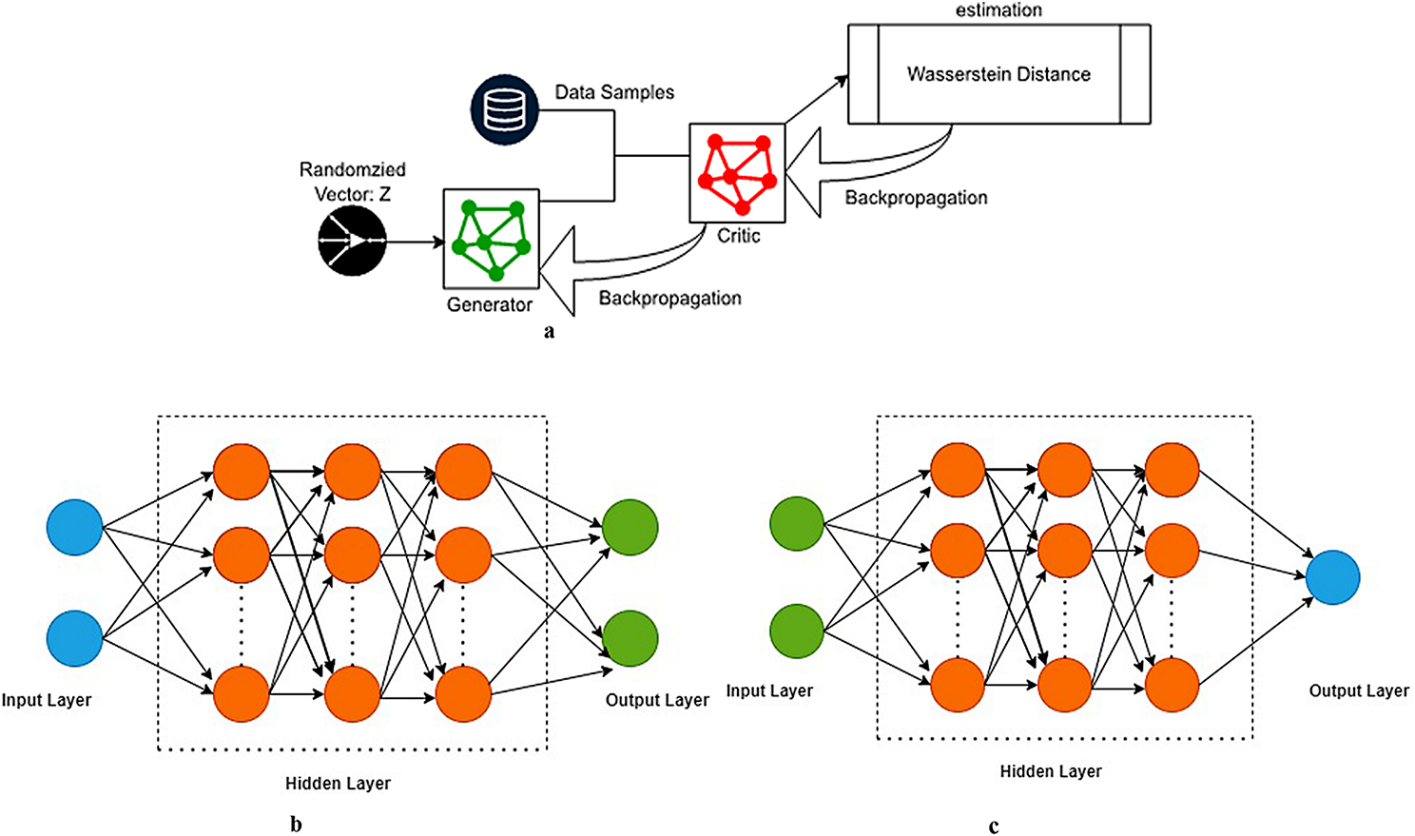
**a** Working Mechanism of WT-GAN. **b** Generator Process. **c**: Discriminator Process

In addition to this, WT-GAN offers a gradient descent (loss function) that has a direct correlation with the veraciousness and accuracy of the generated sample data. It is one of the optimal and potent solutions to GAN degradation (loss). Thus, vanishing gradient and mode collapse are effectively addressed. Since the generated samples are rated rather than classified exactly as true or false, the discriminator is referred to as a critique function [34]. The working mechanism of WT-GAN is shown in Fig. 3a.

#### A. Generator

Training the generator and discriminator simultaneously leads to mode collapse and unstable training. When examining the large quantity of gene expression data, the model needs to produce precise outcomes. The WT-GAN method maintains that the relationships between columns in the real data must be considered to generate synthetic tabular data while keeping data with logical relationships. At the time of backpropagation, the Wasserstein loss value will be calculated to overcome the problem of stable training and mode collapse.

The generator process aims to minimize the objective function, which is shown in Eq. (5). Once the convergence of loss functions of both the generator and critique is done after several pieces of training, the generator begins to produce the synthetic data samples. The generator comprises three layers, which are depicted in Fig. 3b. In the generator process, the inputs are initialized using the he_uniform technique. It selects samples from a uniform data distribution based on the number of weights in the input units. The activators for hidden layers are ReLU, while the activator for the output layer is linear.

5 $$\:\mathbf{min}\left\{\mathbf{log}\varvec{D}\varvec{i}\varvec{s}\left(\varvec{X}\right)+\:\mathbf{l}\mathbf{o}\mathbf{g}(1-\varvec{D}\varvec{i}\varvec{s}(\varvec{G}\varvec{e}\varvec{n}\left(\varvec{Z}\right)\right)\}$$

Where X denotes the real data and Z denotes the fake data generated by the generator network.

#### B. Discriminator

The discriminator process aims to maximize the objective function, which is shown in Eq. (6). Using the GAN’s adversarial approach, the generator sought to create synthetic data samples that might mislead the critique, while the critique intended to distinguish variation among the actual samples as well as the fake ones. To put it another way, a fake sample deceived the critic into thinking it was a genuine one. The finalized synthetic data samples were derived from the initial artificial samples that had deceived the critique network.

6 $$\:\mathbf{max}\left\{\mathbf{log}\varvec{D}\varvec{i}\varvec{s}\left(\varvec{X}\right)+\:\mathbf{l}\mathbf{o}\mathbf{g}(1-\varvec{D}\varvec{i}\varvec{s}(\varvec{G}\varvec{e}\varvec{n}\left(\varvec{Z}\right)\right)\}$$

Where X denotes the real data and Z denotes the fake data generated by the generator network.

Like a generator network, it consists of three layers. The input layer is followed by several hidden layers. Finally, one neuron serves as the system’s output. In the input neurons, the he_uniform technique was implemented. All hidden layers employ the ReLU activator, while the output layer uses the sigmoid activator. The figure depicts an overview of the critic network. The RMSprop optimizer is used to overcome the risk of instability that the Adam optimizer usually poses during training. The binary cross-entropy loss function was used to compute the better accuracy value. Figure 3c depicts the process of building a critic network.

Thus, this guarantees that there will be no gradient disappearing. When the loss function is applied, the produced samples are forced to move closer to the original ones.

### Classification techniques

In this research, traditional machine learning and deep learning methods like Random Forest (RF), Support Vector Machine (SVM), Logistic Regression (LR), Decision Tree (DT), and Feed Forward Neural Network (FFN) are used to classify the different MGECDs after selecting the most significant features [35, 36]. For predicting classification accuracy, the data is divided into 70% for training and 30% for testing in the MGECDs. The different classification techniques were used to predict the samples as “tumor” or “healthy.”

Random Forest builds various decision trees that work together to make a single prediction. It employs a group of classification trees. A bootstrap sample of the data is used to construct each classification tree, and the candidate set of variables at each split is a random subset of the variables. Random Forest builds trees using both random variables and bagging selection. Each tree is grown completely unpruned to create low-bias trees. At the same time, the selection results provide minimal correlation among individual trees. This method generates an ensemble with low bias and low variance.

Support Vector Machine builds the best hyperplane, and it maximizes the distance from both the positive and negative classes. It is used to classify linear as well as nonlinear data. It works based on the threshold value, and it creates a safety margin on both sides to avoid errors. The margin and support vector are used to determine the hyperplane. The support vector is made up of the vectors (data points) that define the hyperplane. The margin is the shortest distance (on two edges) between the nearest point and the hyperplane. When the data is linearly separable, the hyperplane is the line that separates it into two components, each of which belongs to a single class.

Logistic regression is a supervised machine learning technique that can create probabilities and classify new data from both discrete and continuous datasets. It is similar to linear regression, but instead of building the regression line, it builds the logistic curve. It is applied to the classification problem to limit the logistic function, which estimates two maximum values either zero or one.

A Decision Tree is a tree-structured classifier with core nodes representing dataset properties, branches representing decision rules, and leaf nodes reflecting the outcome of the decision. In a decision tree, the algorithm starts at the root node and works its way up to forecast the class of a dataset. The key method underlying a decision tree is the typical top-down divide-and-conquer approach that makes use of the information entropy of different features.

A feed-forward neural network (FFN) is a type of neural network with a hierarchical structure. It consists of an input layer, multi-hidden layers, and a single output layer consisting of several classes. The feed-forward NN receives the initial weights and bias values in the input layer. The neurons are processed and then sent to the output layer via one or more layers that are hidden (h). The hidden layers receive the multidimensional input vector (x) and send it to the output layer (y). It optimizes through backpropagation after processing the inputs with weight and bias (b).

## Results and discussion

### Dataset

The microarray gene expression datasets for colon cancer, prostate cancer, and leukemia cancer are downloaded from the NCBI repository. To test the effectiveness of the strategy, we selected the Microarray Gene Expression Cancer dataset (MGECD) from [37, 38] since it had a massive collection of features composed within two classes that enabled us to evaluate the functioning of the suggested method operated on definite sorts of information. The colon cancer MGECD consists of 2000 features with 62 samples [39]. The leukemia cancer MGECD consists of 72 samples and 3572 features [40]. The prostate cancer MGECD consists of 102 samples and 6033 features [41].

To predict the cancer types based on the MGECD dataset, usually, researchers throughout the globe rely on samples drawn from the databases of prominent bioinformatics labs from world-class universities of advanced learning. Microarray information is widely used in cancer studies in which timely screening of cancer conditions is highly significant in deciding the form of therapy and its prognosis. The datasets contain critical aspects of various cancer maladies, such as colon, leukemia, and prostate cancer. In the medical sector, the term “microarray” is referred to as a research component that can simultaneously measure the interpretation of multiple genes. Diagnosing and categorizing tumor growth using microarray profiling is now universally acknowledged. From the numerous accessible methodologies, there are a handful of enhanced ways to assess microarray information for cancer detection. Additionally, to investigate the proposed approach’s overall impact, we also attempt to compare the accuracy implications of other existing datasets with the microarray gene expression dataset. Several existing datasets were used in various studies but specific to a single cancer disease that is included in Table 2.

**Table 2:** List of datasets

Dataset From	Link	Samples	Features	Disease
[39]	https://www.ncbi.nlm.nih.gov/pmc/articles/PMC7842997 /bin/pone.0246039.s002.csv	62	2000	Colon
[40]	https://www.ncbi.nlm.nih.gov/pmc/articles/PMC7842997 /bin/pone.0246039.s001.csv	72	3572	Leukemia
[41]	https://www.ncbi.nlm.nih.gov/pmc/articles/PMC7842997 /bin/pone.0246039.s003.csv	102	6033	Prostate

**Fig. 4:**
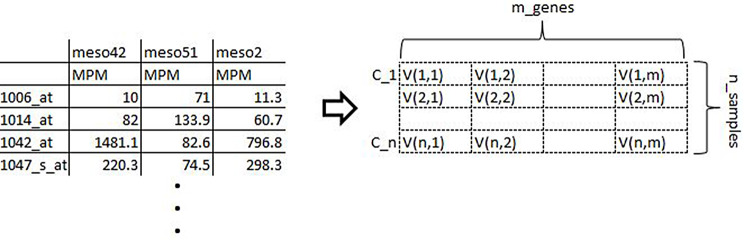
MGECD Tabular Format and its Matrix Form

MGECD is used to exhibit gene expressions that are represented as tables with rows denoting genes (*n*) as well as columns representing samples (*m*) including tumors, normal tissue, or test conditions, where values (*Vpq*) in each block (cell) indicate the level of feature expression of a particular gene () associated with a specific sample [42]. Figure 4 depicts the typical tabular format of MGECD.

### Analysis of correlation-based feature selection

**Fig. 5:**
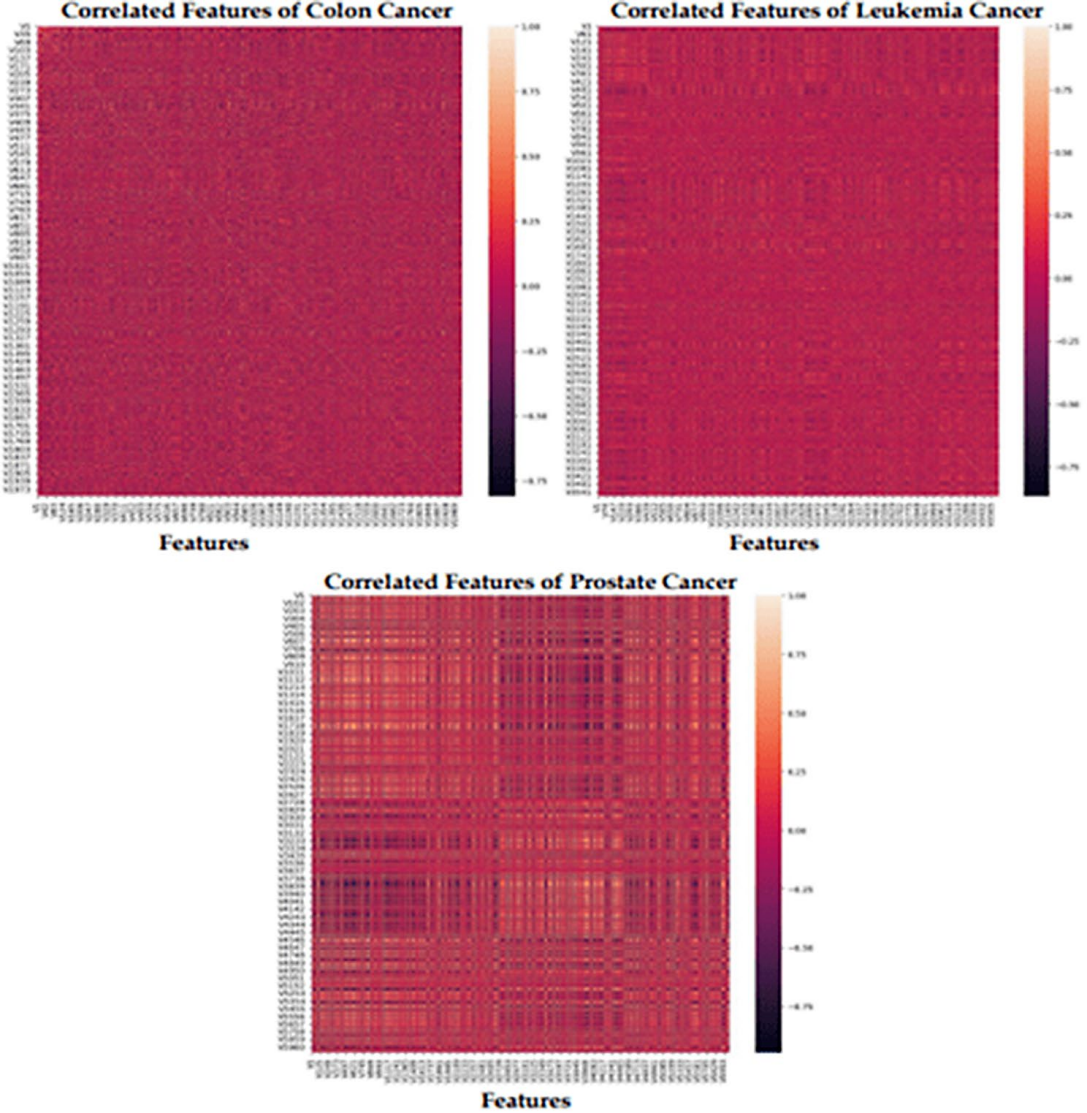
Correlated features of MGECD

The correlation technique is a filter-based approach for retrieving the most relevant genes in the dataset. Choosing the optimal feature set contributes to improved interpretability, lower computing costs, avoiding overfitting, increased accuracy, and better handling of multicollinearity. The objective of the feature selection technique is to remove the related features in the dataset. This technique will work based on the threshold value. To find the correlation between the features initially set the threshold value as zero. Each feature variable is compared with the target variable to find highly correlated features. Figure 5 shows the visualization of correlated each feature for the colon, leukemia, and prostate cancer gene expression datasets using a heatmap.

To retrieve the most relevant features in the gene data, to randomly selected the threshold values, and the performance of the model outcome was good until it reached 1/3 of the feature size. To avoid the overfitting problem, select the relevant features from the original dataset. In colon cancer, 860 features are selected from the 2000 features in the dataset. In this case, 1140 features are correlated if using the threshold value of 0.75. In leukemia cancer, 1329 features are selected from the 3572 features in the dataset. In this case, 2243 features are correlated if using the threshold value of 0.65. In prostate cancer, 1833 features are selected from the 6033 features in the dataset. In this case, 4200 features are correlated if using the threshold value of 0.7. Table 3 shows the selected features and correlated features for different MGECDs based on different threshold values.

**Table 3:** Correlated features

Dataset Name	Number of features	Number of Correlated features	Number of Selected features
Colon	2000	1140	860
Leukemia	3572	2243	1329
Prostate	6033	4200	1833

### Analysis of wasserstein tabular GAN (WT-GAN)

WT-GAN is used to generate the synthesis samples from the original microarray cancer gene expression dataset. The Wasserstein loss value is calculated to reduce the difference between the original and generated data. The proposed method consists of the following steps to define the methods sequentially: (i) To define the method to generate the latent points for the generator. (ii) To define the method for generating the real and fake samples. (iii) To define the method for the generator and discriminator models (iv) To define the WT-GAN model for training and (v) To summarize and evaluate the performance of the proposed model.

The input for the generator is the new latent points in the latent space. For generating the latent points based on the two parameters latent dimension and number of samples. It will randomly generate and reshape the input samples. The generator method is used to generate the fake and real samples with a class label. The parameters for generating the fake samples are generator, latent dimension, and number of samples. The parameter for generating the real samples is the number of samples. To set the class labels while generating the samples to zero for fake samples and one for real samples.

To define the standalone generator model for generating the new samples. The parameters for this model are the latent dimension and the number of outputs. The architecture of the generator network is fully connected with deep neural networks instead of convolutional layers to extract the relevant information in the gene data. This model is executed sequentially in a dense manner. In this model, he_uniform initialization is used, ReLU activations are used in the hidden layers, and linear activation is used in the output layer. To define the standalone discriminator model for discriminating the samples. The architecture of the discriminator network is fully connected with deep neurons instead of convolutional layers to distinguish between the real and fake ones. The parameter for this model is the number of inputs, produced by the generator. This model is executed sequentially in a dense manner. In this model, he_uniform initialization is used, ReLU activations are used in the hidden layers, and sigmoid activation is used in the output layer. This model is compiled with binary cross-entropy, and for optimization, the Adam optimizer is used.

**Fig. 6:**
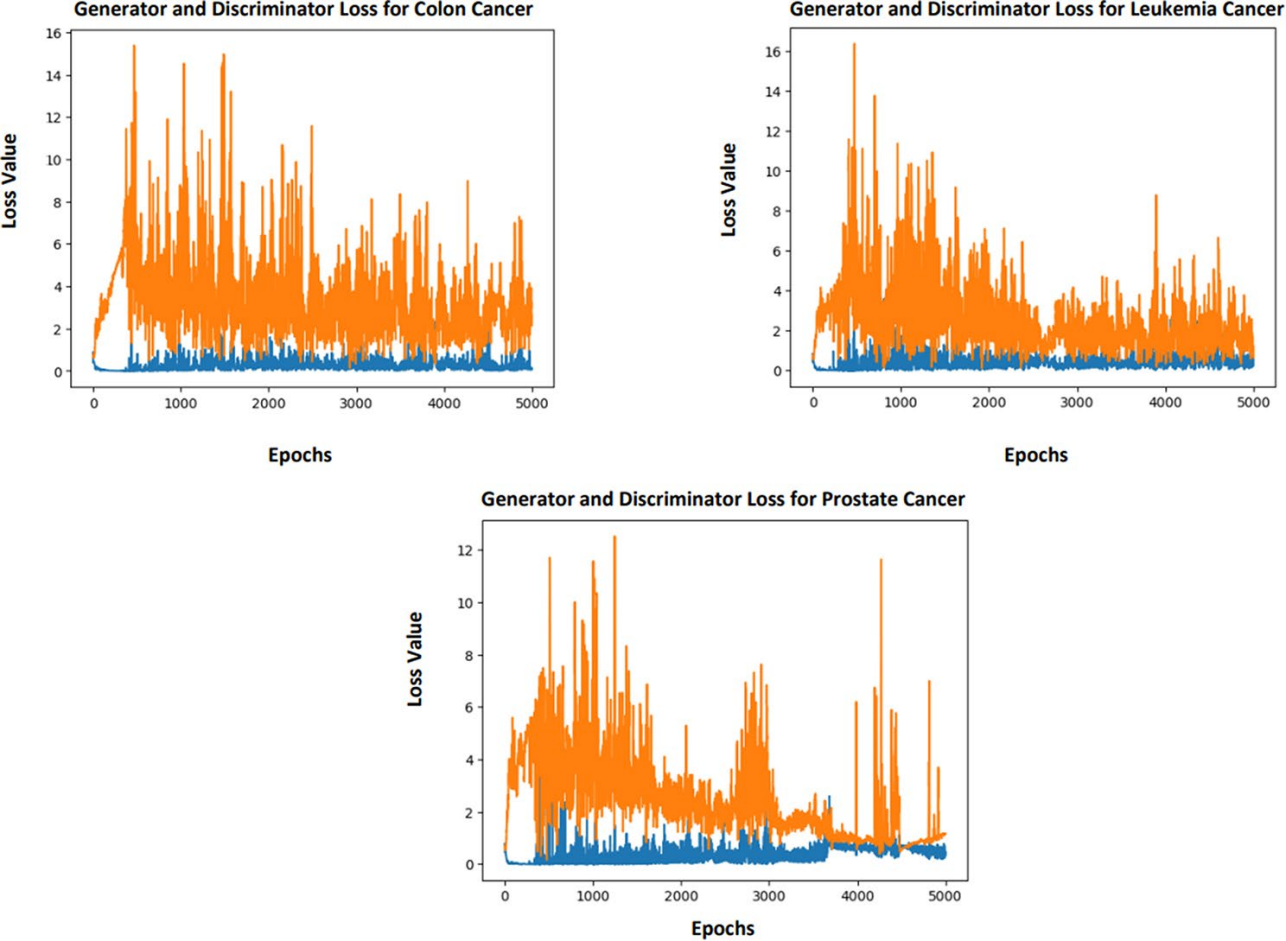
Generator and Discriminator Loss for MGECD

After training all original training samples T_train_ in the WT-GAN Model, the generator network learns the data distribution of each feature in the gene table T and creates the synthetic data table T_synthesis_. The Wasserstein distance is measured by the model to update the loss values. The parameters used for this model are the generator model, discriminator model, latent dimension, number of epochs, and batch size. To update the generator and discriminator loss values, the samples are trained using the proposed model for around 5000 epochs. Then real and fake samples are generated for each epoch based on the half-batch size. First, update the discriminator loss value for real and fake, which has been fixed after training. Then prepare the latent points for the generator based on the latent space for generating the fake samples. To generate the new samples as close as possible to the original samples, update the generator loss based on the discriminator error. Figure 6 show the generator and discriminator loss value for colon, leukemia, and prostate cancer. The generator loss values are plotted in orange, and the discriminator loss values are plotted in blue.

### Performance measures

For critical applications like cancer disease prediction, the influence on performance values was explored about data growth. To increase the sample data size, the WT-GAN approach is utilized. The performance of the proposed predictive model is analyzed in comparison with a few prominent existing systems like RF, SVM, DT, LR, and FNN, which were already discussed in the previous section. One of the significant evaluations of this research begins with data augmentation using Wasserstein Tabular GAN incorporated into the proposed model. The increasing factor of data samples in MGECD is denoted as ‘n’ which influences the accuracy of predictive outcomes. The synthetic data sample becomes more diverse as the size of n rises. It is, obviously, possible to get samples that do not represent the original data if n is too high (significant deviation). On the other hand, if n is maintained at a minimal level, the supplemental data lacks variance. In this work, 100 synthesis samples are generated using the Tabular GAN (T-GAN) and WT-GAN for MGEC datasets. The sample size generated using WT-GAN is compared with the original data (non-augmented data) and T-GAN. The most significant features in the gene data after feature selection are fed into different classifiers like RF, SVM, and FNN. For training purposes, the data is to be split into 70% for training and 30% for testing. Table 4 shows the proposed model performance of MGECD.

**Table 4:** Predictive results for MGECD

MGECD	Classifier	Accuracy			Precision			Recall			F1-Score		
		Original	T- GAN	WT-GAN	Original	T-GAN	WT-GAN	Original	T-GAN	WT-GAN	Original	T-GAN	WT-GAN
**Colon**	RF	0.63	0.89	0.97	0.66	0.87	0.97	0.63	0.86	0.96	0.63	0.86	0.97
	SVM	0.84	0.87	0.93	0.83	0.85	0.86	0.90	0.91	0.98	0.86	0.88	0.92
	FNN	0.68	0.81	0.93	0.72	0.83	0.98	0.69	0.79	0.84	0.70	0.81	0.90
	LR	0.74	0.82	0.96	0.73	0.84	0.97	0.74	0.83	0.98	0.73	0.83	0.97
	DT	0.68	0.83	0.96	0.70	0.86	0.98	0.68	0.85	0.97	0.69	0.85	0.98
**Leukemia**	RF	0.95	0.93	0.97	0.96	0.96	0.98	0.95	0.96	0.98	0.95	0.96	0.98
	SVM	0.92	0.94	0.97	0.81	0.87	0.90	0.73	0.78	0.80	0.82	0.82	0.86
	FNN	0.72	0.84	0.93	0.72	0.84	0.93	0.69	0.81	0.91	0.70	0.82	0.92
	LR	0.91	0.94	0.97	0.91	0.93	0.98	0.90	0.93	0.97	0.93	0.93	0.97
	DT	0.73	0.89	0.97	0.76	0.88	0.98	0.73	0.87	0.90	0.74	0.87	0.93
**Prostate**	RF	0.87	0.92	0.97	0.88	0.91	0.96	0.87	0.92	0.97	0.87	0.91	0.97
	SVM	0.94	0.93	0.97	0.92	0.94	0.98	0.92	0.93	0.97	0.92	0.93	0.96
	FNN	0.80	0.83	0.90	0.75	0.85	0.92	0.85	0.82	0.86	0.80	0.83	0.89
	LR	0.90	0.92	0.97	0.91	0.93	0.98	0.90	0.92	0.97	0.90	0.92	0.98
	DT	0.77	0.84	0.97	0.79	0.85	0.97	0.77	0.86	0.97	0.77	0.85	0.97

According to the findings from Table 4, the data augmentation strategy using WT-GAN produces better classification results when compared to non-augmented data (original data) and augmented data with T-GAN. The T-GAN can give better outcomes when compared to the original data. The WT-GAN achieved better results when compared to the T-GAN and original data. For critical applications like cancer disease prediction if the data samples are insufficient then the proposed augmentation strategy gives better results when compared to existing approaches.

The proposed method’s efficiency is evaluated with traditional tabular GAN data and non-augmented data in terms of classification accuracy, recall, precision, and F1 score. For evaluating the performance of four measures, the following four parameter values are required. The true positive (TP) refers to the predicted values being positive and the actual values being positive as well. The true negative (TN) refers to the predicted values being negative and the actual values being negative as well. The false positive (FP) refers to a type-I error where the predicted values are positive but the actual values are negative. The false negative (FN) refers to a type-II error where the predicted values are negative but the actual values are positive.

The classification accuracy can be measured by the percentage of the total number of samples that are correctly classified as positive and negative, divided by the total number of samples. Equation (7) represents the formula for the accuracy value.

7 $$\:\mathbf{A}\mathbf{C}\mathbf{C}\:=\:(\mathbf{T}\mathbf{P}+\mathbf{T}\mathbf{N})\:/\:(\mathbf{T}\mathbf{P}+\mathbf{T}\mathbf{N}+\mathbf{F}\mathbf{P}+\mathbf{F}\mathbf{N})$$

The sensitivity value is also known as the true positive rate or recall value. It can be measured by the percentage of the number of samples correctly classified as positive, divided by the total number of positive samples in the actual class. Equation (8) represents the formula for the recall value.

8 $$\:\varvec{R}\varvec{e}\varvec{c}\varvec{a}\varvec{l}\varvec{l}=\mathbf{T}\mathbf{P}\:/\:(\mathbf{T}\mathbf{P}+\varvec{F}\varvec{N})$$

The positive predicted value is also known as the precision value. It can be measured by the percentage of the number of samples correctly classified as positive, divided by the total number of positive samples in the predicted class. Equation (9) represents the formula for the precision value.

9 $$\:\mathbf{P}\mathbf{r}\mathbf{e}\mathbf{c}\mathbf{i}\mathbf{s}\mathbf{i}\mathbf{o}\mathbf{n}\:=\mathbf{T}\mathbf{P}\:/\:(\varvec{T}\varvec{P}+\varvec{F}\varvec{P})$$

The F-Score value is also known as the F-Measure. It is a single measure of the classification procedure’s usefulness and it can be measured by the harmonic mean of precision and recall value. Equation (10) represents the formula for the F1-Score value.

10 $$\:\varvec{F}-\varvec{S}\varvec{C}\varvec{O}\varvec{R}\varvec{E}=2\:\mathbf{*}\:\left(\varvec{P}\varvec{r}\varvec{e}\varvec{c}\varvec{i}\varvec{s}\varvec{i}\varvec{o}\varvec{n}\mathbf{*}\varvec{R}\varvec{e}\varvec{c}\varvec{a}\varvec{l}\varvec{l}\right)\:/\:(\varvec{P}\varvec{r}\varvec{e}\varvec{c}\varvec{i}\varvec{s}\varvec{i}\varvec{o}\varvec{n}+\varvec{R}\varvec{e}\varvec{c}\varvec{a}\varvec{l}\varvec{l})$$

**Fig. 7:**
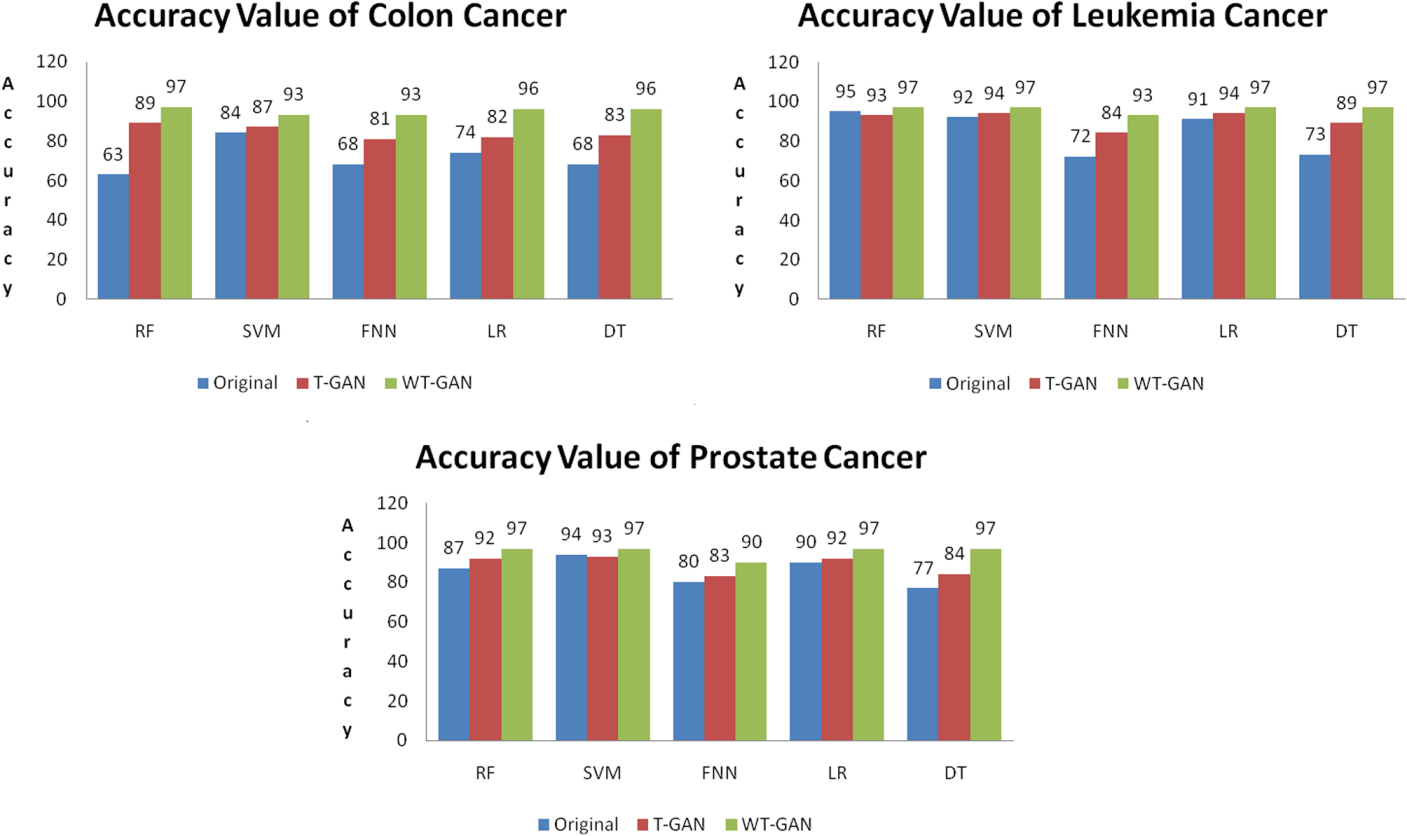
Accuracy value obtained from MGECD

Figure 7 exhibits the accuracy value obtained from the non-augmented data (original data), T-GAN, and proposed WT-GAN. It clearly shows that the proposed technique achieved better classification accuracy for colon, leukemia, and prostate gene expression data. The WT-GAN model achieved a value of around 97% accuracy for the colon, leukemia, and prostate datasets. The proposed model accuracy results are compared with the existing paper [10], the colon, leukemia, and prostate cancer achieved 91.7%, 88.4%, and 93.6%, respectively. The proposed WT-GAN augmentation strategy improves the classification accuracy of 5.3%, 8.6%, and 3.4% against traditional tabular GAN. The observed accuracy value for colon cancer is 96% in the paper [24] using an RF classifier, and the leukemia cancer is 87.88% in the paper [25]. The proposed model achieved 97% accuracy in predicting cancer disease.

**Fig. 8:**
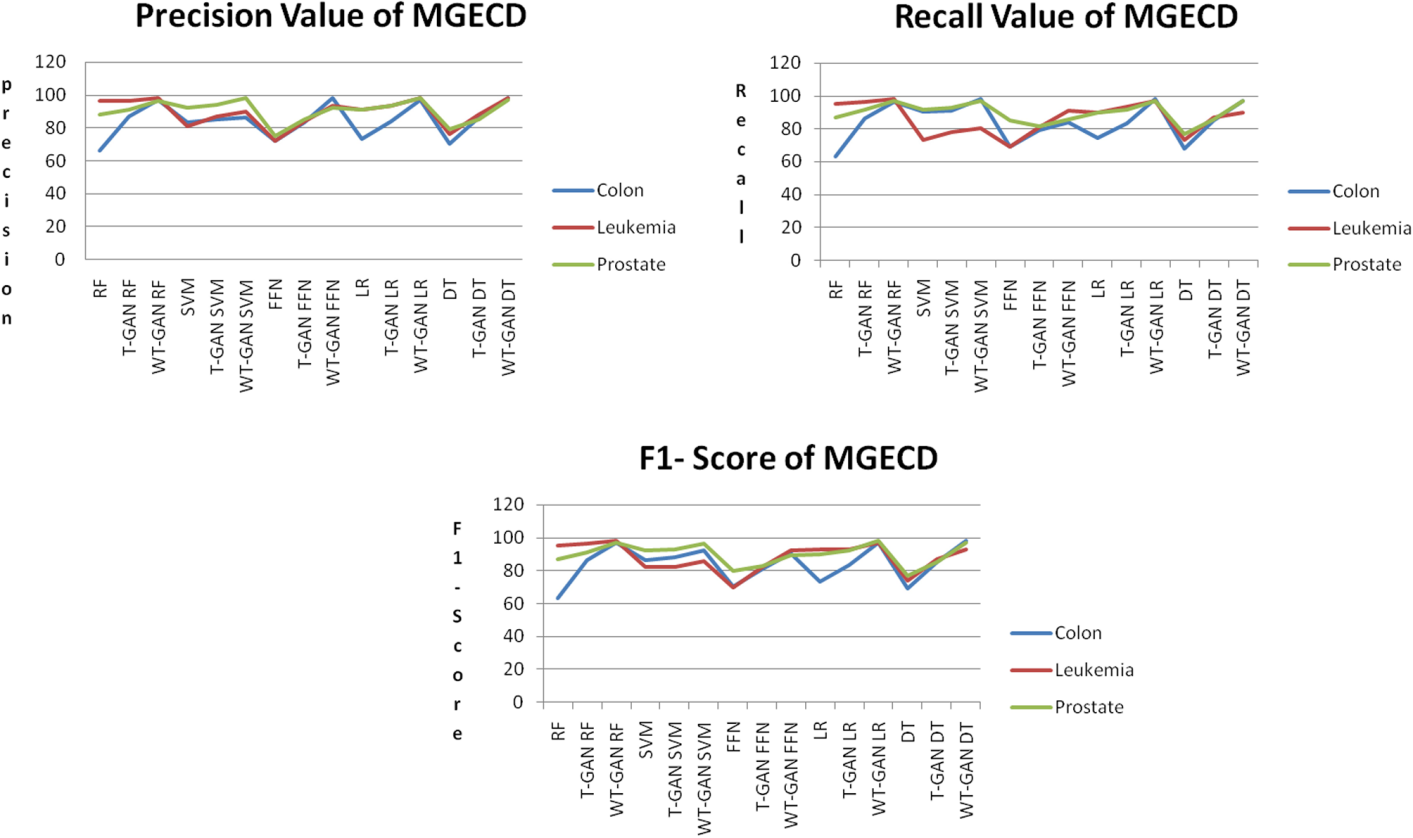
Precision, Recall, and F1-Score Value of MGECD

Figure 8 depicts the precision, recall, and F1-score values for MGECD. The proposed augmentation method WT-GAN, improved the precision, recall, and F1-Score value when compared to T-GAN and original data. For colon cancer, the FFN and DT achieved 98% precision, and the SVM and LR achieved 98% recall value. For leukemia cancer, the RF, LR, and DT achieved 98% precision, and the RF achieved 98% recall value. For prostate cancer, SVM and LR achieved 98% precision, and the RF, SVM, LR, and DT achieved 97% recall value. Furthermore, the observed sensitivity value using the RF classifier is a 0.4% variation from the existing paper [22]. In tabular data augmentation, the proposed model WT-GAN can enhance better outcomes.

### Evaluation of the quality of tabular data

**Fig. 9:**
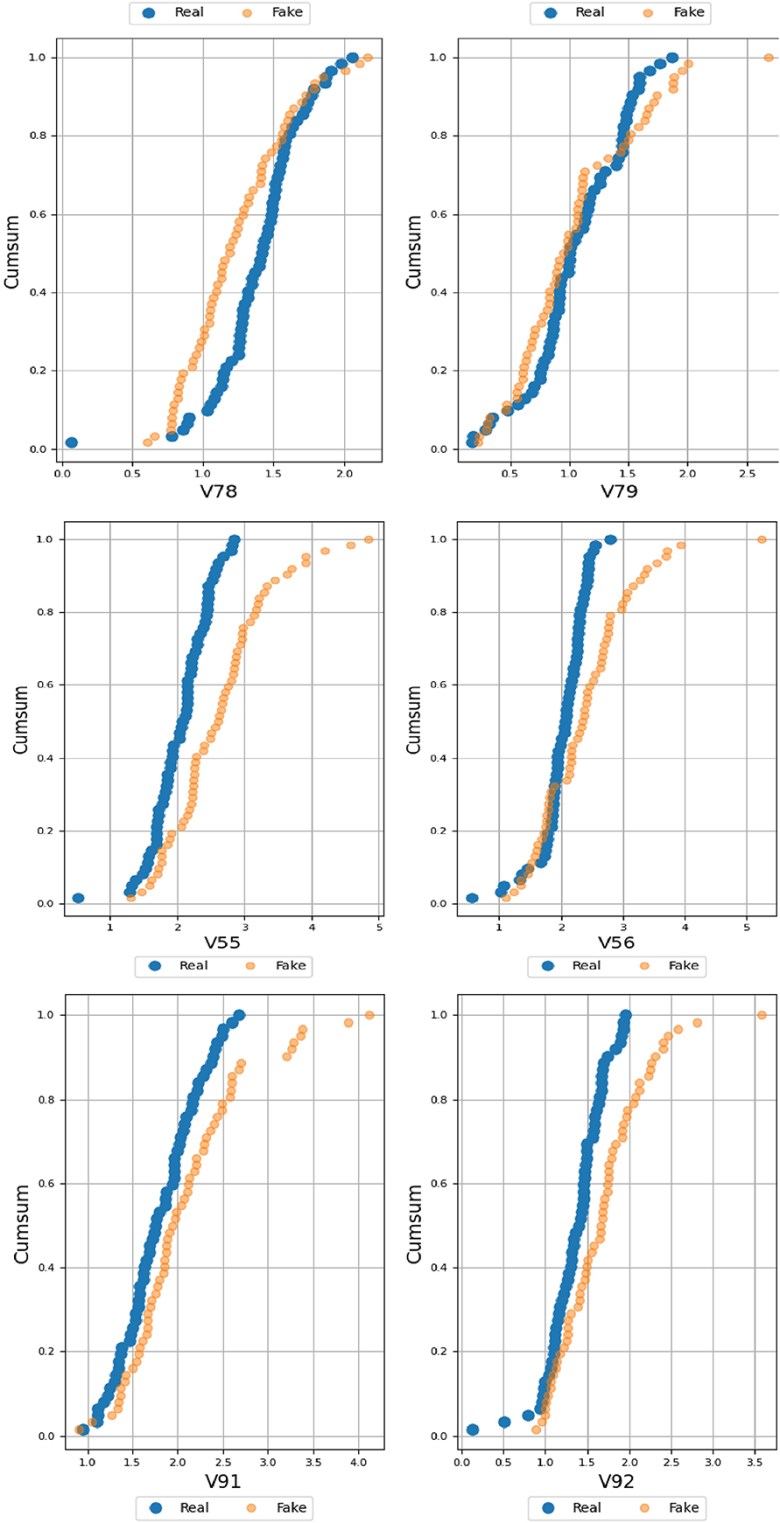
Similarity between real and fake data of MGECD

To evaluate the quality of fake data and real data, a table evaluator is used. The table evaluator is a library for determining how similar a fake dataset is to a real dataset. It is appropriate for evaluating the synthetic data created. Many applications are becoming possible with the growth of GANs, which have been designed for tabular data. It shows the similarity between the data distribution between the real and fake ones. Figure 9 shows the similarities between the real and fake data. It indicates that fake distributions are in orange and real distributions are in blue.

## Conclusion

Studies have proven that researchers need a lot of information to draw a sensible conclusion when it comes to making critical decisions. Applications in the medical field need a large amount of data to draw results that can be trusted. MGECD was found to be unbalanced when there were fewer samples with a high number of characteristics. Because gathering more data samples in the experimental conditions is a laborious process, this point insists the researchers generate artificial data using the advanced methodology. For serious examination, the artificially produced data must be closely related to the original data. WT-GAN along with correlation feature selection is used to generate the data in this research work.

According to the findings, the Wasserstein loss function with tabular data augmentation is utilized. The loss value is significantly reduced to create the synthetic samples close to the original samples. As a result, the generated data was guaranteed to be sensitive. Accuracy, recall, and precision were used to assess the classifier’s effectiveness. Furthermore, the ultimate random forest, logistic regression, decision tree, support vector machine, and feed-forward neural network classifiers are used. The augmented data had the highest accuracy of the techniques studied, reaching ~ 97% accuracy. The findings show that increasing the generator’s performance will significantly improve the prediction performance. As a result, data generation for critical applications like cancer prediction might benefit from tabular data augmentation. The generator in the proposed model uses high-dimensional data space to learn from the training samples, which is a shortcoming. Increasingly large samples allow researchers to refine their focus on finding the optimal combination of features.

## Data Availability

Data is provided within the manuscript.
